# Psychological Determinants of Physical Activity and Development in Early Childhood Among Children With Developmental Delays: The Role of Parent Beliefs Regarding the Benefits of Physical Activity

**DOI:** 10.3389/fspor.2020.00104

**Published:** 2020-08-18

**Authors:** Kimberley D. Lakes, Ross D. Neville, Maryam Abdullah, Joseph Donnelly

**Affiliations:** ^1^Department of Psychiatry and Neuroscience, School of Medicine, University of California, Riverside, Riverside, CA, United States; ^2^Department of Pediatrics, School of Medicine, University of California, Irvine, Irvine, CA, United States; ^3^School of Public Health, Physiotherapy, and Sports Science, University College Dublin, Dublin, Ireland; ^4^Greater Good Science Center, University of California, Berkeley, Berkeley, CA, United States; ^5^Center for Autism and Neurodevelopmental Disorders, School of Medicine, University of California, Irvine, Irvine, CA, United States

**Keywords:** neurodevelopmental disorders, developmental disabilities, autism, physical activity, parent beliefs, BMI, psychological determinants

## Abstract

Among the various psychological determinants of physical activity (PA) in early childhood, relatively little attention has been paid to the role of parent beliefs in the benefits of PA for their child. Believing that PA is beneficial may impact parent behavior, resulting in more opportunities for PA in early childhood, particularly among children with neurodevelopmental disabilities (NDs) who may face more barriers to PA. Greater opportunity for PA may promote the development of motor skills and healthy body composition. This study examined the association between parental beliefs about PA and children's weight status in a sample of 147 children (32 ± 4 months old) with NDs. The proportion of parents with below average (mean − 1SD) perceptions of the benefits of PA whose children were overweight or obese was approximately was 2.5 times (proportion ratio, 2.35; 95% CI, 1.05–5.27) larger than it was for parents with above average (mean + 1SD) perceptions (after adjusting for the confounding effects of ethnicity, marital status, and mothers' self-reported depressive symptoms). Mothers' self-reported depressive symptoms was the only other covariate that was significantly associated with the weight status of these children, though, these data also signal possible (*p* = 0.07) differences in proportions between Hispanic/Latinx and White children in the sample who were classified as overweight and obese. Our study demonstrates the importance of considering parental or caregiver beliefs in the value of PA as another risk factor that may predict risk for overweight and obesity. Future studies should include parental beliefs in the benefits of PA as a potential psychological determinant of PA and associated health outcomes.

## Introduction

The International Classification of Functioning, Disability and Health (ICF: World Health Organization, 2002) describes a biopsychosocial model of human functioning and disability that provides a highly relevant framework for studying the psychological and behavioral determinants of physical activity (PA). From the ICF perspective, functioning or activity is impacted by interactions between health conditions and contextual factors (environmental and personal). This perspective is particularly useful for understanding the relationships between neurodevelopmental disabilities, PA, and psychological and physical health. For children with neurodevelopmental disabilities (NDs), difficulties related to attention, communication, self-regulation, and motor control are common and may become barriers to participation in social and recreational activities, including activities that provide opportunity for PA (e.g., Must et al., 2015; Lakes et al., 2019a), which is essential for psychological, social, and physical development. Barriers may include personal factors, such as children's emotional and behavioral difficulties (e.g., Law et al., 2007) as well as environmental factors, including the need for more supervision and trained providers in recreational programs (e.g., Must et al., 2015). Reductions in opportunities for PA can, in turn, lead to increased sedentary time. For example, children (ages 8–18) with Autism Spectrum Disorder (ASD) have been reported to average 62% more screen time than their typically developing peers (Mazurek and Wenstrup, 2013; American Psychiatric Association, 2013). While this increased screen time is likely due to a number of factors, facing personal and environmental barriers to physically active social and recreational activities may contribute to the amount of time spent in sedentary activities.

These patterns of activity have implications for mental and physical health. Children with NDs or other developmental disabilities have an increased risk of obesity and overweight when compared to typically developing children (e.g., Curtin et al., 2010; Broder-Fingert et al., 2014; Zuckerman et al., 2014; Hill et al., 2015; Zheng et al., 2017), which may compound disability and reduce quality of life (e.g., Khodaverdi et al., 2011; Dhaliwal et al., 2019). Reduced PA and increased sedentary behavior are among the developmental, biological, and environmental risk factors for overweight among children with NDs (Dhaliwal et al., 2019). Therefore, a better understanding of the psychological and behavioral determinants of PA as well as the relationship between patterns of activity and overweight are important research priorities for this population.

Because children with NDs may face more barriers to PA (such as the need for extra supervision on the playground or the need to find PA programs equipped to support their behavioral, motor, and social challenges), strong parental support for PA and strong parental beliefs in the benefits of PA are likely to be especially critical. Supporting PA in the face of many personal and environmental barriers may require substantial commitment to and belief in the importance of PA. Lakes et al. (2017) described how parental beliefs play an important role in the development of positive health behaviors in children and hypothesized that parental beliefs about the benefits of PA are likely to influence parent behavior, particularly the degree to which parents seek and maintain opportunities for their child with ND to engage in PA. The association between such parental support and observed levels of PA in children has been documented by Seibert et al. (2016) who found that parent support and a parent's perceptions of his/her child's physical abilities predicted PA in children with disabilities. Similarly, Brown et al. (2020) found that among parents of school-age children with ASD, parent intentions to support PA were associated with parent PA support behaviors, which in turn predicted PA in children. Their research illustrates the important role parents play in promoting PA among children with NDs.

Our goal in this study was to examine potential psychological determinants, particularly those that might be modifiable via early intervention, that might contribute to PA patterns in young children with NDs as well as the trend toward overweight and obesity in this population. While PA is not the sole predictor of healthy weight in children, it is considered a protective factor against the risk for overweight and is also a modifiable factor that could be addressed in many settings. Given the important role parents play in promoting PA (e.g., Brown et al., 2020), in this study, we explore the association between parental beliefs in the value of PA and weight status in children with NDs. In doing so, we estimate the degree to which differences in parental beliefs about the benefits of PA predict differences in the proportions of children with NDs classified as overweight and obese, whilst also considering as potential confounders other social and environmental factors that have been associated with body composition (and health disparities) in prior research (e.g., child race and ethnicity, household income, cognitive functioning, adaptive behavior, and sleep patterns).

## Materials and Methods

### Participants and Procedures

This study was approved by a University Institutional Review Board. Over a 1 year period, 147 new patients (between the ages of 24 and 39 months at the time of their evaluation) scheduled for or seeking new diagnostic evaluations at a university neurodevelopmental clinic were enrolled in this study. All prospective patients meeting the age criteria who were either scheduled for or requesting a new diagnostic evaluation during the 12 month study time period were invited to participate in the study. Inclusion criteria were: (1) between the ages of 23–39 months at enrollment, and (2) scheduled for or seeking a new diagnostic evaluation at the center for any developmental concern. Participants were allowed to enroll at age 23 months, but all visits took places when the child was between 24 and 39 months of age. Among the 158 eligible children whose parents were contacted, 147 (93%) enrolled in the study. Several declined participating in research, and several others declined because they chose not to follow through with their appointments at the university center either because they had received care elsewhere or had concerns about insurance coverage for university services. The university neurodevelopmental clinic where the study took place was a specialty center for NDs in Southern California, and at the time served as the primary ND center for a county with a population of more than 3 million people.

A parent or legal guardian provided written informed consent for their child's participation, which included three study visits. During the first two visits, parents completed questionnaires, and clinical researchers administered psychological measures, including Module 1 or 2 of the Autism Diagnostic Observation Schedule (ADOS; Lord et al., 2012). During the third visit, a physician (a pediatric neurologist or developmental behavioral pediatrician) evaluated the child and met with the parents. Children who (1) met the criteria for the cutoffs “autism” and “autism spectrum” on the ADOS and (2) were diagnosed with Autism Spectrum Disorder (ASD) by a physician were noted as having ASD (*n* = 118), which was the most common diagnostic category among participants. Other diagnoses included global developmental delay (*n* = 63, with some overlap with ASD), speech and language disorders or delays (*n* = 69, with some overlap with ASD and global developmental delay), Attention Deficit Hyperactivity Disorder (ADHD: *n* = 1), hypotonia (*n* = 1), cerebral dysfunction (*n* = 2), and disruptive behavior disorder (*n* = 2).

### Measures

#### Diagnosis of Autism Symptoms

The Autism Diagnostic Observation Schedule (ADOS; Lord et al., 2012) was used to evaluate autism symptoms. A psychology postdoctoral fellow and two board certified developmental behavioral pediatricians who were trained in ADOS administration conducted the ADOS evaluations. Either Module 1 or Module 2 was administered, based on the child's language development. Scores yield a classification of either non-spectrum, Autism spectrum, or Autism.

#### Primary Dependent Variable

Children's Body Mass Index (BMI) was obtained using the measured height and weight data at the time of their study physician evaluation. BMI percentile and weight status category were calculated using the Centers for Disease Control and Prevention's (CDC) BMI Percentile Calculator (Centers for Disease Control, 2015).

#### Primary Independent Variable

The Parent Perceptions of Physical Activity Scale (PPPAS) is a 25-item parent-report questionnaire (Lakes et al., 2017) available in two forms—one for infants (Lakes et al., 2019b) and the second for young children (Lakes et al., 2017), which was used in this study. The PPPAS requires about 10 min to complete and assesses parental perceptions of the benefits of (18 items) and barriers to (7 items) PA, using a 4-point Likert scale with responses ranging from Strongly Disagree (1) to Strongly Agree (4). Sample items for the Benefits subscale included: “Increasing activity increases my child's level of physical fitness,” “Physical activity in childhood will make my child healthier,” “Exercising helps my child sleep better at night,” and “Physical activity improves overall functioning for my child.” Sample items for the Barriers subscale included: “I worry that my child will not be accepted by others if he/she participates in a group sport or activity program” and “Physical activity will make my child frustrated.” Principal components analysis (PCA) for the 25 items yielded two components (Lakes et al., 2017): (1) beliefs in the benefits of physical activity and (2) perceptions of barriers to physical activity. The PPPAS was initially developed for and studied in children under the age of five who were experiencing developmental difficulties as a tool to assess parental perceptions that might impact their PA support behaviors. Validity and reliability of PPPAS scores in this population (children ages 2–3 years with NDs) were reported by Lakes et al. (2017); scores demonstrated good (Cronbach's alpha = 0.83 for Barriers to PA) to excellent (Cronbach's alpha = 0.95 for Benefits of PA) internal consistency as well as sufficient concurrent, discriminant, and predictive validity.

#### Covariates

The Vineland Adaptive Behavior Scales, Second Edition (Vineland-II; Sparrow et al., 2005) was used to evaluate children's adaptive skills, including communication, daily living skills, socialization, and motor skills. Responses yield standard scores based on United States norms. The manual reports excellent internal consistency coefficients. Among 3 year-old children in the standardization sample, the split-half reliability coefficients were 0.92, 0.90, 0.95, 0.90, and 0.97 for the Communication Domain, Daily Living Skills Domain, Socialization Domain, Motor Skills Domain, and Adaptive Behavior Composite scores, respectively. Test-retest reliability coefficients for ages three to six were 0.92, 0.91, 0.88, 0.90, and 0.94 for the Communication Domain, Daily Living Skills Domain, Socialization Domain, Motor Skills Domain, and Adaptive Behavior Composite scores, respectively. Vineland II scores also showed excellent validity for individuals with ASD (Sparrow et al., 2005), and the measure has been widely used in ASD research.

Parents also completed a brief study-specific demographic questionnaire, a self-report depression screening that measures depressive symptoms over the prior week (Patient Health Questionnaire-9: Kroenke et al., 2001) and a child sleep questionnaire (Children's Sleep Habits Questionnaire: Owens et al., 2000). Children's Sleep Habits Questionnaire scores have shown adequate internal consistency in prior research with clinical populations (internal consistency coefficient = 0.78) as well as adequate test-retest reliability (correlations from 0.62 to 0.79 for the various subscales: Owens et al., 2000).

Parents completed a brief physical activity questionnaire that asks parents to report on the amount of time a child spent engaged in a variety of physical activities over the course of the prior week (Weekly Physical Activity Checklist: Sallis et al., 1993). The instructions indicate that only activities in which the child engaged for more than 15 min at a time should be included. Twenty-eight activities are listed, with space to write in additional activities. Sample activities included: outdoor play (including playground structures), bike riding, swimming, water play, games (including tag), ball sports, walking, running/jogging, martial arts, dancing. For this study, we calculated a total score for weekly bouts of PA of all types. These checklists have been used in numerous studies involving children of different ages, though we are not aware of prior studies validating this measure in 2 and 3 year-old children with NDs. However, many of the listed activities were deemed relevant for this population (e.g., outdoor play, water play, playing with balls, walking, and running), and activities that were not relevant could be simply ignored by the respondent. Any additional activities could be written in. Responses were recorded using checkmarks across the 7 days of the week.

A psychology postdoctoral fellow assessed cognitive functioning using the Mullen Scales of Early Learning (Mullen, 1995). The Mullen contains a battery of tests designed to measure development in infants and preschoolers. Four cognitive scales (receptive language, expressive language, visual reception, fine motor) are used to generate a standardized Early Learning Composite score. The Early Learning Composite score is considered an early measure of overall cognitive function. In the standardization sample (Mullen, 1995), the split-half reliability coefficient for the Early Learning Composite Score in 33–38 month-old children was excellent (0.92). Among children ages 25 to 56 months, test-retest reliability coefficients for the Mullen scales were sufficient, ranging from 0.71 to 0.75. Interscorer reliabilities among 25 to 44 month-old children were excellent (0.98 to 0.99).

### Statistical Analyses

The association between parental perceptions of the benefits of PA and the likelihood of overweight and obesity in children was analyzed with a logistic regression model in the Statistical Analysis System (SAS: Version 9.4; SAS Institute, Cary, NC). To explore this association, we first categorized children into four groups based on their level of BMI: underweight (BMI below the 5th percentile); healthy weight (BMI between the 5 and 85th percentile); overweight (BMI between the 85 and 95th percentile); and obese (BMI > 95th percentile). Once categorized, a binomial distribution was used to model the odds of overweight and obesity, and to compare the proportions of children classified as overweight or obese for parents with above (mean+1SD) and below (mean−1SD) average perceptions of the benefits of PA. The research question was answered by estimating the ratio of proportions of children classified as overweight or obese for parents with high and low perceptions of the benefits of PA. Or, put more simply as a question, was the proportion of children classified as overweight or obese substantially lower for parents with above average beliefs in the benefits of PA? The answer to this question was estimated with other covariates held constant at their mean values.

When presenting the results, proportions were chosen over odds due to the time-independent nature of the classification and due to the high incidence of overweight and obesity in children (Greenland, 1987; Nurminen, 1995). Research shows that odds and incidence proportion ratios are only approximately equal to one another when the proportions in comparison groups is uncommon or rare (<10%) (Tamhane et al., 2016). Therefore, presenting the results as proportions avoids the well-documented issues of overestimation that have been associated with the reporting of odds ratios (Greenland, 1987; Nurminen, 1995; Tamhane et al., 2016). Confidence limits for the proportion ratios were derived from the confidence limits for the odds. Magnitude thresholds for a factor increase and decrease in the proportion ratio were as follows: for an increase, 1.1 = small, 1.4 = moderate, 2.0 = large, 3.3 = very large, 10 = extremely large; for a decrease, 0.9 = small, 0.7 = moderate, 0.5 = large, 0.3 = very large, 0.1 = extremely large (Hopkins et al., 2009). Uncertainty in the estimate is expressed as a 95% confidence interval.

#### Handling of Missing Data

Table 1 provides descriptive statistics of complete cases for our primary outcome variable and covariates. It also identifies where there were missing values in our data. Research has shown that there is substantial bias in odds ratio estimates (and, consequently, in proportion ratio estimate which are derived from the odds) when the percentage of missing data is >5% (Knol et al., 2010). Since the percentage of missing data was >5% for both the primary dependent and independent variable, our results represent data analyzed by means of multiple imputation (Berglund and Herringa, 2014). Because the missing values had a monotonic pattern, the logistic and linear regression methods were used for imputing values for categorical and continuous variables, respectively. We used the imputation diagnostics and variance information to estimate the number of imputed datasets required to ensure adequate efficiency (i.e., a relative efficacy score above 0.99, where a score of 1 equals perfect efficiency). We followed an iterative process, making reference to the formula for the Fraction of Missing Information (FMI) (Rubin, 1987), which summarizes the proportion of sampling variance in the model that is due to missing data: [*V*_*B*_+ (*V*_*B*_/*m*)]/*V*_*W*_, where V_B_ and V_W_ are the estimated variances between and within imputed datasets, respectively, and where m represents the number of imputations specified at this iteration. The imputation procedure is deemed to have had acceptable efficiency when the number of imputations (m) specified results in an FMI of <1%. In the present study, 26 imputed datasets were required to ensure adequate efficiency.

**Table 1:** Socio-economic-, parent-, and child-level sample characteristics.

**Characteristic**	**Value**
**Socio-economic factors**	
Ethnicity, No. (%)	
White	105 (73)
Latinx	39 (27)
Missing	3 (2)
Household income, No. (%)	
< $20,000	19 (13)
$20,000–$30,000	13 (9)
$30,000–$50,000	12 (8)
$50,000–$75,000	25 (17)
$75,00–$100,000	24 (16)
>$100,000	40 (27)
Missing	14 (10)
**Parent-level factors**	
Married, No. (%)	105 (73)
Missing	(3) (2)
Parent's age, mean (SD) [No. missing]	35.34 (7.54) [16]
Number of children, mean (SD) [No. missing]	2.25 (1.20) [3]
Parental depression, mean (SD) (0–27) [No. missing]	3.92 (4.52) [14]
PPPAS, benefits of PA, mean (SD) (1–4) [No. missing]	3.56 (0.41) [22]
PPPAS, barriers to PA, mean (SD) (1–4) [No. missing]	1.73 (0.55) [15]
**Child-level factors**	
Age in months, mean (SD) [No. missing]	31.66 (4.37) [3]
Sex, No. (%)	
Boy	106 (72)
Missing	3 (3)
BMI, mean (SD) [No. missing]	16.59 (1.72) [11]
BMI percentile No. (%)	
Underweight: <5th percentile	11 (7.5)
Healthy weight: 5th−85th percentile	94 (64)
Overweight: 85–95th percentile	16 (11)
Obese: >95th percentile	15 (10)
Missing	11 (7.5)
Total weekly PA in bouts of activity, mean (SD) [No. missing]	17.21 (12.00) [5]
Sleep habits^a^, mean (SD) [No. missing]	47.10 (8.88) [28]
Daytime sleepiness, mean (SD) (8–24) [No. missing]	11.74 (2.85) [10]
Adaptive behavior^b^, mean (SD) [No. missing]	81.44 (13.00) [4]
Cognitive functioning^b^, mean (SD) [No. missing]	66.94 (18.14) [7]

a *T score (mean: 50 [SD: 10])*.

b *Standard score (mean: 100 [SD: 15])*.

*No., number; SD, standard deviation; PPPAS, Parent Perceptions of Physical Activity Scale; PA, physical activity; BMI, body mass index; TV, television*.

## Results

### Participant Characteristics

Demographic and subject characteristics for the 147 parents (age, mean ± SD: 35 ± 8 years; 82% mothers) and children (32 ± 4 months) involved in this study are reported in Table 1. The majority of parents were married, had multiple children, above- and below-average self-reported health and depressive symptoms (respectively), and were running households with annual income levels (slightly positively skewed) toward the upper end of the distribution. Parents also had well above average perceptions of the benefits of PA, with more moderate perceptions about the absence of barriers. The majority of children were boys (approximately three-quarters) and were White (more than two-thirds). Parental reports of children's daily screen-viewing time, sleep habits, and daytime sleepiness were approximately average. Parental reports also indicated that children were engaging multiple meaningful bouts (15 min or more) of PA across the week (on average, 2–3 bouts per day). Based on standard scores, children were ranked well within the lower tertile of the normal distribution for adaptive behaviors and cognitive functioning.

### Preliminary Analyses

Preliminary analysis was conducted according to Bursac et al. (2008) process for the purposeful selection of covariates in a logistic regression model. The initial set of covariates considered for inclusion in the logistic regression model were as follows: parental perceptions of the benefits of PA, parental perceptions of the barriers to PA; child's sex, age, sleep habits, levels of adaptive behavior, cognitive functioning, and frequency of engagement in weekly PA; family race and ethnicity and household income; and mothers' age, marital status, and self-reported depressive symptoms. The first step in the process of purposeful selection involved univariate analyses of the predictive value of each individual covariate against a significance threshold of *p* < 0.25. Only covariates meeting this initial inclusion threshold of *p* < 0.25 were considered for further multivariate analysis. Covariates were thereafter retained in the final logistic regression model if they fulfilled either of the following two criteria: (i) the covariate itself had a substantial effect magnitude and significance threshold of *p* < 0.10, or (ii) removal of the covariate had a substantial confounding effect on the magnitude of other remaining covariates' parameter estimates (reflected by a ≥ 15% change in the predictive value of another covariate). Following this iterative model-building process resulted in the following four variables being retained in the final logistic model predicting proportions of overweight and obesity: parental perceptions of the benefits of PA, ethnicity, marital status, and mothers' self-reported depressive symptoms. Contrary to our expectations, parent reports of a child's weekly PA did not meet criteria for inclusion in the final model.

### Primary Analyses

Based on imputed values for BMI percentile, in the final analysis, 11 children were underweight, 94 were healthy weight, and 31 were overweight or obese. As for our primary research question, the association between parental perceptions of the benefits of PA and the proportion of children who are classified as overweight and obese is summarized (alongside other covariates) in Table 2. The proportion of parents with below average (mean−1SD) perceptions of the benefits of PA whose children were overweight or obese was approximately was 2.5 times (proportion ratio, 2.35; 95% CI, 1.05 to 5.27) larger than it was for parents with above average (mean+1SD) perceptions (after adjusting for the confounding effects of ethnicity, marital status, and mothers' self-reported depressive symptoms). When viewed against the thresholds for evaluating factor effect magnitudes outlined in the methods section above, this difference in proportions represents a large magnitude (i.e., a factor difference of >2).

**Table 2:** Associations between covariates and the proportion of children classified as overweight and obese.

**Characteristic**	**Value^a^**	**95% CI**	***p*-value**
**Socio-economic factors**			
Ethnicity^b^	1.74	0.95 to 3.17	0.07
**Parent-level factors**			
Married^b^	0.69	0.42 to 1.14	0.15
Parental depression^c^	0.41	0.18 to 0.90	0.03
PPPAS, benefits of PA^c^	2.35	1.05 to 5.27	0.04

a *Values are proportion ratios, adjusted for the confounding effect of other covariates*.

b *Values represent factor differences in the proportion of overweight and obese children associated with a difference of level (e.g., comparing White and Latinx children, or comparing children of married and unmarried mothers)*.

c *Values represent factor differences in the proportion of overweight and obese children associated with a two standard deviation (SD) difference in the predictor (e.g., comparing the proportion of overweight and obese children in parents with above [mean+1SD] and below [mean−1SD] average perceptions of the benefits of PA)*.

*CI, confidence interval; PA, physical activity*.

Of the other covariates that were included in the final model (see Table 2), only mothers' self-reported depressive symptoms was statistically significantly associated with the proportions of children classified as overweight and obese. Perhaps counterintuitively, the proportion was ~2.5 times smaller (proportion ratio, 0.41; 95% CI, 0.18 to 0.90) for children of parents with above average (mean+1SD) self-reported depression. Marital status was not a statistically significant predictor of overweight and obesity in this sample of children. However, when considered against Bursac et al.'s (2008) criteria for inclusion of a covariate (see section Preliminary Analyses above), its removal from the model resulted in a substantial modification of the parameter estimate for maternal depression (signaling shared variance between marital status and mothers' self-reported depressive symptoms). Finally, whilst ethnicity was not statistically significantly associated with weight status in the final model, with a *p*-value of 0.07, it is noteworthy that the proportion of Hispanic or Latinx children that were classified as overweight and obese was ~1.75 times larger (proportion ratio, 1.74; 95% CI, 0.95 to 3.17) than the proportion for White children. *Post-hoc* analysis revealed that the association between parental perceptions of the benefits of PA and the proportion of children who are classified as overweight and obese did not differ significantly between Hispanic/Latinx and White children (proportion ratio, 0.84; 95% CI, 0.40 to 1.78).

## Discussion

Prior research has suggested that parent beliefs and behaviors may be psychological determinants of PA in children with disabilities. In research with 148 parents of children with disabilities (Seibert et al., 2016), found that parent support for PA and beliefs in their child's perceived competence for PA predicted their promotion of PA. Our overarching hypothesis was that parental beliefs in the benefits of PA would influence the degree to which a parent supports or encourages engagement in PA for young children with NDs, and that this would be reflected in early risk for overweight. Our study provides preliminary support for this hypothesis and extends prior research by documenting an association between parental beliefs in the benefits of PA and child BMI. The proportion of parents with above average (mean+1SD) perceptions of the benefits of PA whose children were overweight or obese was 8%. The proportion was 4 times higher for parents with below average (mean−1SD) perceptions. In other words, approximately one third of the sample of children whose parents had below average perceptions of the benefits of PA were overweight or obese.

A parent's report of perceived barriers to PA was not a substantial predictor of BMI. It is also worth noting that, in this relatively young sample (age, mean ± SD: 32 ± 4 months), the mean score for a parent's perceived barriers to PA was 1.73 on a 4.0 scale, where a score of “2” represents “disagree.” Thus, on average, parents in this sample tended not to agree with items measuring barriers (such as perceptions that their child may not have the skills needed to engage in PA or that others might reject their child in a group PA setting). The types of weekly PA reported in this sample primarily included outdoor play, walking, and swimming, with only a few parents reporting that their children were bike riding, playing sports, or engaging in other organized recreational activities. It is likely that personal (e.g., motor competence) and sociocultural barriers to physically active social and recreational activities (including those addressed in the PPPAS) become more salient as children age, and this issue should be addressed in samples of older children with NDs, as a parent's perception of a child's competence (i.e., a potential personal barrier) was found to predict promotion of PA in prior research (Seibert et al., 2016). It is also likely that the PPPAS does not capture all environmental, sociocultural, and personal barriers to PA experienced by children with NDs in this age range. In future research, qualitative methods (i.e., interviews, focus groups) could be used to identify additional personal (e.g., parent stress, parent-child relationships, child behaviors) and environmental barriers (e.g., social attitudes toward children with NDs, availability of accessible programs and facilities) to engagement in PA for young children with NDs.

Parents' self-reported depressive symptoms were the only substantial and statistically significant confounder. For the association between parent depressive symptoms and child overweight, the proportion was 2.5 times smaller (proportion ratio, 0.41; 95% CI, 0.18 to 0.90) for children of parents with above average (mean+1SD) self-reported depression (after adjusting for the confounding effects of ethnicity, marital status, and parental perceptions of the benefits of PA). In other words, parents with above average depressive symptoms were 2.5 times less likely to have children who were overweight or obese. In a recent review of research examining the associations between maternal depression and child overweight, Lampard et al. (2014) found that chronic, but not episodic, depression was positively associated with child overweight. The PHQ-9 used in our research captures current depressive symptoms (over the last 2 weeks) and does not distinguish between episodic depression or chronic depression; however, our results were in the opposite direction of what would be expected for chronic depression and were not consistent with the null finding for episodic depression. Our results also contrasted with those noted in a large study with 4,601 5th grade children, wherein researchers documented a positive association between maternal depression (i.e., symptoms experienced in the prior 7 days), single parenthood, and child BMI, and noted that parenting quality and its relation to sedentary behavior and leisure activity mediated the association (McConley et al., 2011). It is possible that the differences in our findings might differ from other literature as a result of sample characteristics, including the very young age of the children and their developmental difficulties and clinical characteristics. Moreover, our analysis also revealed the possibility of an interaction effect between parent depression and marital status. *Post-hoc* analyses of our data revealed a significant correlation between these two variables, with currently married parents reporting fewer depressive symptoms (*r* = −0.252, *p* = 0.005). Researchers have documented an increased prevalence of depressive symptoms among mothers of children with ASD, particularly around the time of first diagnosis (Taylor and Warren, 2012), so it may be that different patterns of depressive symptoms in this population explain these divergent findings. It's important to note that our study was conducted at the time of first diagnosis, when depressive symptoms may be more prevalent as well as potentially more transient; mothers, particularly single mothers, facing a new diagnosis of an ND in their 2–3 year old child may exhibit a different pattern of maternal depression than has been studied in prior research examining the relationship between maternal depression, beliefs about PA, and child overweight and obesity.

Finally, it is worth noting that, based on BMI percentile, 11 children were underweight, 94 were healthy weight, and 31 (21% of the sample) were overweight or obese. Prior research (Broder-Fingert et al., 2014) indicated that the risk for obesity was higher in older children (ages 12–15) compared to younger children (ages 6–11) with ASD, but there is limited epidemiological research on patterns of overweight in toddlers and preschoolers with ASD. The Centers for Disease Control in the United States (CDC: Hales et al., 2017) estimates that 13.9% of two- to five-year-old children in the United States are obese; in our sample of children (age, mean ± SD: 32 ± 4 months), 15% were obese and another 16% were overweight. A direct comparison between these statistics is difficult as the CDC estimate includes a much wider age range and focused on obesity; future research should examine the point at which the trajectory of overweight for children with NDs begins to diverge from that of children without NDs, as prior research has suggested that the risk for overweight and obesity grows with age in this vulnerable population (e.g., Broder-Fingert et al., 2014; Hill et al., 2015). Finally, ethnicity approached significance in the model, and Hispanic or Latinx children were 1.7 times more likely than White children to be overweight or obese. These results are consistent with epidemiological studies showing higher rates of unhealthy weight in Hispanic children (Hales et al., 2017) and illustrate the importance of studying health disparities, environmental risks, or differential susceptibility for overweight in early childhood to better understand the factors associated with this increased risk.

### Study Limitations and Future Directions

While the results of this study extend prior research and contribute to our knowledge about factors associated with unhealthy weight in early childhood among children with NDs, different patterns could be observed in different age groups. Future research should, therefore, assess the relationship between parental perceptions of the importance of PA and childhood overweight in a sample of children with NDs across a broader age range. Parental influence may be less salient when other risks (e.g., psychiatric medications) are introduced or become more established (e.g., restricted food interests, sedentary behaviors such as screen time). The current study also was somewhat limited in its ability to examine racial and ethnic disparities in childhood overweight and obesity, given the demographics in the region in which the study was conducted. Future research should examine these relationships in populations who experience health disparities. In addition, the majority of participating parents in this study were mothers (82%), and our sample was not large enough to examine differences between mothers, fathers, or alternative guardians (e.g., custodial grandparents). Future research should examine how these associations are similar and different based on the caregiver's relationship to the child and influence on the child's daily patterns of activity.

Parental reports on a child's weekly engagement in PA were not a significant predictor of overweight in this study, but this research should be replicated with the inclusion of objective measures of PA (e.g., accelerometers), as parental report of PA is known to have some recall limitations. In addition, as very young children may engage in more frequent or shorter bouts of PA, it is possible that the measure's focus on bouts of 15 min or more may have limitations in this age group. These limitations could be addressed in future research by using a range of measures, including parent report measures and objective measures of activity.

Finally, our study was limited in that we were not able to collect data on other variables known or hypothesized to impact child overweight, including data on genetics, nutrition, maternal metabolic disorders, endocrine dysregulation, or gut microbiota diversity. Dhaliwal et al. (2019) reviewed developmental, biological, and environmental factors associated with weight gain in children with Autism Spectrum Disorder (ASD), categorizing them as either primary risk factors (those that have been directly implicated to weight gain or obesity), secondary risk factors (factors not specific to children with ASD but which could adversely affect weight), and emerging factors (factors the authors hypothesize may play a role). Primary risk factors included genetics and the use of psychiatric medications for which weight gain is a side effect. Secondary risk factors included parent obesity, nutrition, PA, and sedentary behavior. Emerging risk factors included breastfeeding, maternal metabolic disorders, sleep, endocrine dysregulation, and gut microbiota diversity. Most of the research reviewed by Dhaliwal et al. focused on older children, who have already established patterns of behavior surrounding nutrition, screen time, physical activity, and psychiatric medication use. Our study was designed to contribute to prior research by focusing on a very young group of children, who were not yet taking psychiatric medications and who, due to their young age, had less firmly established behavioral health patterns. Therefore, in spite of limitations due to the exclusion of biological and nutritional factors, our results controlled for a number of socioeconomic, parent, and child factors, and our results suggest that future studies should consider parent beliefs about PA as an important psychological factor to consider when evaluating the diverse risks for overweight in early childhood.

### Implications for Parent Education in Interventions, Educational, and Clinical Settings

One of the implications of our findings is that strengthening parental beliefs in the value of PA for young children with NDs may help contribute to healthy development and body composition. Parent education programs could incorporate information about the positive effects of PA on multiple domains of development and functioning. The importance of PA for physical growth, development, and health is well-established. However, the benefits of PA extend beyond direct physical benefits. Research describing the positive effects of physically active social and recreational programs has grown exponentially in the last 15 years. Physically active interventions that involve cognitive engagement are associated with improvements in executive functions [e.g., see reviews and meta-analyses by Diamond and Lee (2011); Vazou et al. (2016), and Takacs and Kassai (2019)] and self-regulation [e.g., for a review see Pandey et al. (2018)], although substantial variation between different types of interventions has been documented. Children with NDs often exhibit difficulties with executive functioning and self-regulation, suggesting that activities that promote these skills may be especially relevant for their development. Physically active interventions have also shown a positive effect on academic achievement [see review by de Greeff et al. (2018)], another area that is often a concern for children with NDs. Thus, for children with NDs, PA interventions may have the potential to positively impact psychological and academic functioning in addition to physical health.

Research reporting positive effects of physically active interventions for children with NDs, including ASD, is still emerging (Srinivasan et al., 2014), but preliminary studies have shown positive outcomes, such as reductions in stereotypical behaviors (e.g., Ferreira et al., 2019; Lakes et al., 2019a). Of particular salience to early childhood is recent research in a large Irish cohort study (Neville et al., 2020) that demonstrated that for boys with early developmental delay, involvement in sports is associated with a significant decrease in behavioral difficulties between ages 3 and 5 years. This suggests that organized PA in the preschool years could potentially improve behavior even at this early age and is especially promising as it demonstrated this positive effect among boys with developmental delay.

Beginning at first diagnosis of an ND and throughout childhood, clinicians should address parental beliefs about the benefits of PA for their children. Sharing evidence for the positive effects of PA on psychological and physical development may increase a parent's beliefs that PA can be beneficial, which may in turn influence parental behaviors associated with seeking and maintaining opportunities for their children to be active. Future research should examine the degree to which increasing parental perceptions of PA as beneficial are directly associated with increased engagement in PA as well as increased parental behaviors associated with seeking and maintaining opportunities for PA. Developing strong PA habits at an early age could contribute to better long-term health outcomes for children with NDs, and parental beliefs in the benefits of early PA may help support the development of these positive habits.

## Data Availability Statement

The raw data supporting the conclusions of this article will be made available by the authors, without undue reservation.

## Ethics Statement

The studies involving human participants were reviewed and approved by University of California, Irvine. Written informed consent to participate in this study was provided by the participants' legal guardians.

## Author Contributions

KL, MA, and JD: study concept and design and study supervision. RN and KL: analysis and interpretation of data and drafting of manuscript. KL, RN, MA, and JD: critical revision of manuscript for important intellectual content. KL: obtained funding. All authors: contributed to the article and approved the submitted version.

## Conflict of Interest

The authors declare that the research was conducted in the absence of any commercial or financial relationships that could be construed as a potential conflict of interest.
